# Anatomy guided modality fusion for cancer segmentation in PET CT volumes and images

**DOI:** 10.1038/s41598-025-95757-6

**Published:** 2025-04-09

**Authors:** Ibtihaj Ahmad, Sadia Jabbar Anwar, Bagh Hussain, Atiq ur Rehman, Amine Bermak

**Affiliations:** 1 Northwestern Polytechnical University, Xi’an, 710072 Shaanxi People’s Republic of China; 2 School of Public Health, Shandong University, Jinan, Shandong People’s Republic of China; 3 Division of Information and Computing Technology, College of Science and Engineering, Hamad Bin Khalifa University, Doha, Qatar

**Keywords:** Cancer imaging, Machine learning

## Abstract

Segmentation in computed tomography (CT) provides detailed anatomical information, while positron emission tomography (PET) provide the metabolic activity of cancer. Existing segmentation models in CT and PET either rely on early fusion, which struggles to effectively capture independent features from each modality, or late fusion, which is computationally expensive and fails to leverage the complementary nature of the two modalities. This research addresses the gap by proposing an intermediate fusion approach that optimally balances the strengths of both modalities. Our method leverages anatomical features to guide the fusion process while preserving spatial representation quality. We achieve this through the separate encoding of anatomical and metabolic features followed by an attentive fusion decoder. Unlike traditional fixed normalization techniques, we introduce novel “zero layers” with learnable normalization. The proposed intermediate fusion reduces the number of filters, resulting in a lightweight model. Our approach demonstrates superior performance, achieving a dice score of 0.8184 and an $\hbox {HD}^{95}$ score of 2.31. The implications of this study include more precise tumor delineation, leading to enhanced cancer diagnosis and more effective treatment planning.

## Introduction

PET offers metabolic information that helps to identify and assess malignancies such as tumors. In PET scans, the tumor typically exhibits a higher standardized uptake value. Therefore, PET scans are crucial for clinical diagnosis and radiotherapy treatment. However, PET lacks anatomical information and has low resolution. CT on the other hand, offers a high-resolution view of anatomical structures. However, CT does not reveal much about malignancies. In conjunction, the oncologists use PET to examine lesions and CT to find the anatomical context. Combining these modalities improves the segmentation performance^1^. Given that PET-CT modalities are in the form of volumes, automated analysis with computer assistance is still difficult because of the redundant data in these enormous volumes.

Early Computer-Aided Diagnosis (CAD) systems used individual 2D PET or CT slices to train deep-learning segmentation models. However, because they do not take 3D information into account, they produce low-quality segmentation^2^. These segmentations can not be used for critical procedures, such as radiotherapy. 2.5D methods were introduced to use 3D information at a lower computational cost^3^. However, 2.5D methods still use part of the 3D volumes, resulting in lower-quality segmentation. With the development of powerful Graphical Processing Units, more accurate 3D approaches have become possible. These methods produce better segmentation at the expense of more resources. These methods use PET or CT separately or combine them to produce better results^4,5^. The effective techniques for combining these modalities are based on early or late fusion^6,7^. However, these methods cannot independently learn complementary features of CT and PET modalities. Furthermore, PET and CT images are less correlated at the volume level and should not be fused as channels^1^. On the other hand, late fusion techniques utilize more resources and cannot use shallow inter-modality features.

We resolve these issues via anatomy guided intermediate attentive fusion mechanism. We excite the encoders with individual modalities through the squeeze and excite method to improve the quality of spatial representation. We then fuse PET features with CT features guided by anatomical attention. We demonstrate that our approach reduces the number of filters required, making the model lightweight. We also add a “zero layer,” which has a learnable normalization layer. Furthermore, we train and evaluate the model with volumes and images (2D slices). The following are the major contributions of this work:

- We extract features from each modality separately via excited encoders to improve the spatial representations and then fuse only useful features intermediately through a guided attentive fusion mechanism.
- We propose zero layers, which use learnable normalization. The learnable normalization produces significant improvements compared to the fixed normalization used by the state-of-the-art.
- Compared to the state-of-the-art, our model is less complex in terms of training parameters. Furthermore, unlike the state-of-the-art models, the suggested model applies to volumes and slices.

## Related work

The development of deep learning models significantly improved automatic computer-assisted diagnosis. The earliest deep learning techniques outperformed the conventional image segmentation methods^8–10^. These techniques have more generalization capability yet possess huge improvement gaps. The performance gaps are improved with the UNet style encode-decode structures^11–15^. These techniques works well for images, however for applications like radiotherapy planning, 2D segmentation they are unreliable because it is susceptible to discontinuity in 3D space^2^. Powerful Graphical Processing Units made the development of 3D models possible which with higher computational costs, can capture 3D features^2,16^. Early 3D models utilized single-modality encoder-decoder architecture.^17^ proposed the first encoder-decoder style model to segment tumors in CT images.^18^ used a 3D UNet with deformable convolution blocks. Other variants of UNet are proposed to improve the segmentations^19–21^. In contrast to encoder-decoder style methods,^22^ suggested a 3D FCN generate a probability map from the modality and segment the tumor from its surrounding soft tissues.^23^ suggested a multi-resolution network to integrate features from different image resolutions and feature levels via residual connections.^24^ integrated 2D dense connection CNN with 3D VNet. These techniques only use one modality, which yields unsatisfactory segmentation and is inadequate for applications involving complicated medical procedures, e.g., radiation therapy^25^.

PET and CT modalities are fused to achieve superior segmentation performance^20,26^. These methods are classified as late fusion and early fusion. Late fusion, such as the one proposed by^7^ and^27^, generally employs distinct CT and PET models combined at the decision level. Late fusion models require a lot of resources^6^. In contrast, early fusion methods fuse CT and PET at the modality level^28^. Early fusion-based methods, such as^29^ and^30^, use attention mechanisms to exploit the advantages of spatial attention. The early fusion method based on 3D-Inception ResNet employs modified Inception units as encoders, while ResNet units as decoders^31^. Early fusion-based methods are often less resource-hungry and outperform late fusion techniques in segmentation performance^6^. However, early fusion-based methods have drawbacks, i.e., they lack the ability to learn the complementary features among each modality^7^. Furthermore, CT and PET modalities are less correlated at the volume level^1^. Intermediate fusion strategies combine the benefits of both methods while overcoming their drawbacks^32–34^. These methods have demonstrated improved performance for segmenting 2D medical images. However, they rely on massive intermediate connections. 3D volumes, such as PET and CT, drastically increase the required intermediate connections due to the depth of these modalities. By reducing the massive number of features caused by the depth of modalities via some mechanism, the intermediate fusion can become less resource-hungry while producing improved results. According to recent literature, the fusion via attention mechanism is more effective than other methods^35^. Fusion via the attentive mechanism is now being used in other image processing applications^36,37^. These techniques have outperformed other intermediate fusion techniques in terms of performance. However, designing fusion via the attentive mechanism is challenging in the case of PET and CT^38,39^. Since both modalities are in the form of huge 3D volumes, designing complex intermediate fusion mechanisms may increase the computational burden. Another important factor that can highly affect the segmentation in PET and CT is the normalization^40,41^. PET scans produce images where pixel intensities represent tracer uptake, often expressed as Standardized Uptake Values (SUVs). These values can vary significantly depending on the patient body weight, injected dose, and scanner calibration. Similarly, with a defined range, CT scans measure tissue density in Hounsfield Units (HU). These values might be consistent across scans but differ substantially from PET values. Both of these modalities need to be normalized. Existing methods rely on fixed normalization, which might not accurately normalize if the scans are from different sources. These fixed methods might include standard slipping, z-score normalization, sine wave, etc.^19,20,42,43^. To solve the above mentioned problems, this work aims to design an intermediate fusion mechanism that includes learnable normalization and may require fewer resources.

**Fig. 1:**
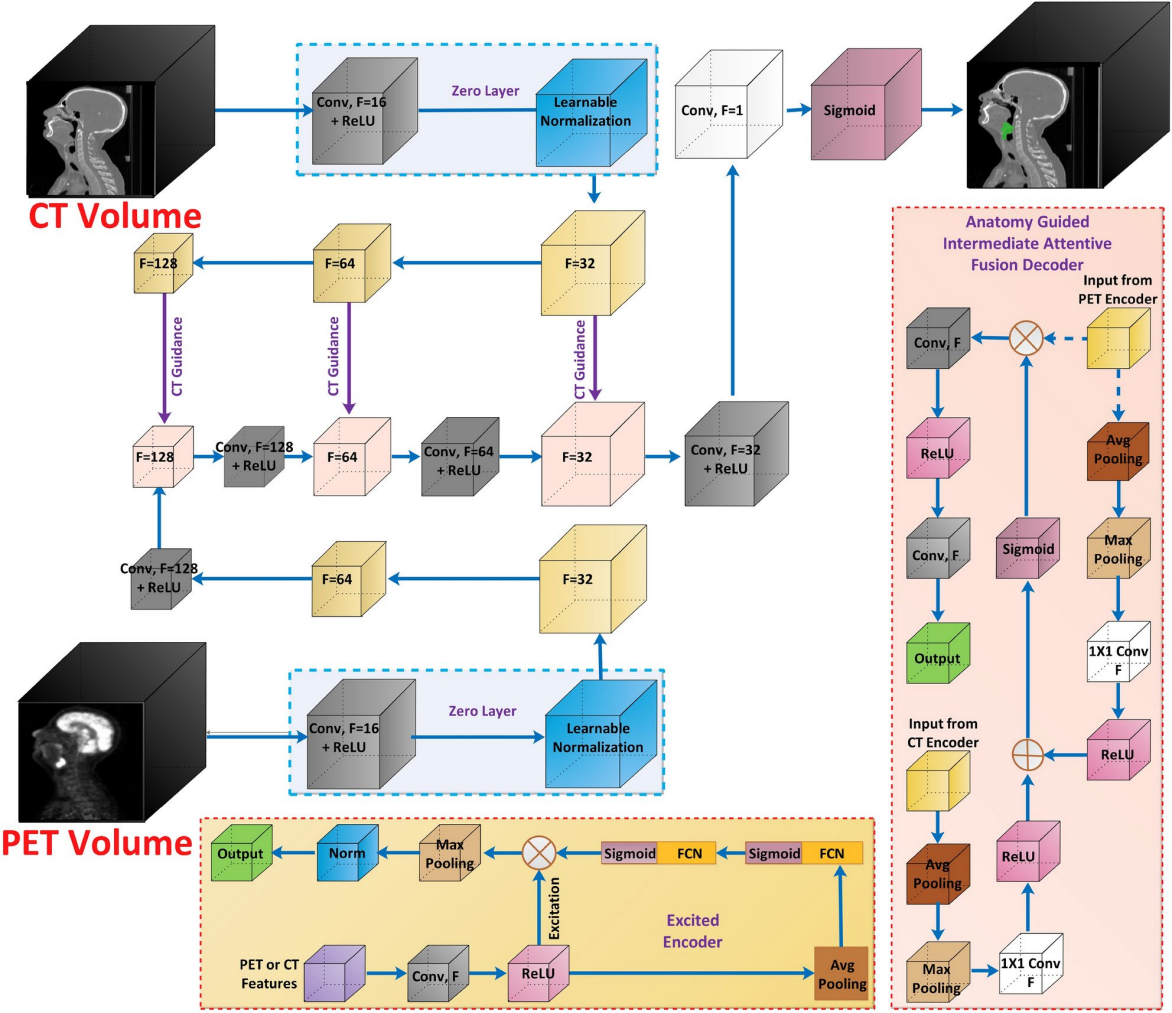
The figure illustrates our architecture along with the excited encoder and anatomy guided intermediate fusion decoder..

## Proposed architecture

The proposed architecture uses learnable normalization layers for each modality to learn the normalization for each input modality. The architecture uses parallel squeeze and excited (SE) encoders for PET and CT features. These encoders extract relevant features from each modality separately. The encoders are followed by an anatomy-guided intermediate fusion mechanism (Fig. 1) via the specially designed decoders. Finally, it outputs the semantic segmentation mask of the cancer. The major components of the architecture are explained in detail as follows:

### Zero layers

The model has two inputs for each modality (*V*) which is independently passed through a “zero layer”. The learnable normalization layer at this stage plays a significant role in performance improvement since the normalization of CT and PET is essential due to the variations caused by capturing environment and equipment. Although we perform normalization at the pre-processing stage (refer “Datasets” for details), this secondary normalization adds to the performance improvement. The improvement caused by this additional normalization is verified in the ablation study (refer “Ablation studies”).

The model takes PET and CT volumes as inputs, denoted as $M_{\text {PET}}$ and $M_{\text {CT}}$, respectively. Each volume passes through the *Zero Layer*. The zero layers consist of convolution (*Conv*) with the kernel (*k*) and filters (*F*), followed by ReLU ($\rho$) and normalization (*N*) layers, respectively. The normalization is learnable via gamma ($\lambda _{PET/CT}$), beta ($\delta _{PET/CT}$) parameters, and a learnable constant $\epsilon$.

$$\begin{aligned} P_{\text {PET}}= & \rho \left( Conv^F_{k,k}(M_{\text {PET}})\right) \\ P_{\text {CT}}= & \rho \left( Conv^F_{k,k}(M_{\text {CT}})\right) \end{aligned}$$

The normalized outputs for PET and CT are given by:

$$\begin{aligned} & Norm (\text {PET}) = \lambda _{\text {PET}} \cdot \left( \frac{P_{\text {PET}} - \mu (P_{\text {PET}})}{\sqrt{\sigma ^2(P_{\text {PET}}) + \epsilon }}\right) + \delta _{\text {PET}} \\ & Norm (\text {CT}) = \lambda _{\text {CT}} \cdot \left( \frac{P_{\text {CT}} - \mu (P_{\text {CT}})}{\sqrt{\sigma ^2(P_{\text {CT}}) + \epsilon }}\right) + \delta _{\text {CT}} \end{aligned}$$

### Squeeze and excited encoders

PET and CT carry complementary information but differ significantly in the kinds of features they emphasize. The squeeze-and-excite mechanism allows the model to adaptively emphasize the most important features from each modality. By learning weighting, the model can decide how much importance to give to each feature map based on the specific modality, thus improving the overall feature representation.

The normalized outputs from zero layers are processed through their respective encoders, which consist of convolution layers (Each stage of encoder have different number of filters, i.e. $F=[128, 64, 32, 16]$) and ReLU activations ($\rho$).

$$\begin{aligned} Enc_{\text {PET}}= & \rho \left( Conv^{F}_{3,3}(Norm_{\text {PET}})\right) \\ Enc_{\text {CT}}= & \rho \left( Conv^{F}_{3,3}(Norm_{\text {CT}})\right) \end{aligned}$$

Then the global features $g$ are extracted via average pooling is:

$$\begin{aligned} g_{\text {PET}}= & Pool_{\text {GAP}}(Enc_{\text {PET}}) \\ g_{\text {CT}}= & Pool_{\text {GAP}}(Enc_{\text {CT}}) \end{aligned}$$

The excitation is calculated by passing $g$ through fully connected layers with ReLU and sigmoid activations ($\sigma$):

$$\begin{aligned} Exc_{\text {PET}}= & \sigma (FCN(\rho (FCN(g_{\text {PET}})))) \\ Exc_{\text {CT}}= & \sigma (FCN(\rho (FCN(g_{\text {CT}})))) \end{aligned}$$

The final excited features are computed by modulating the fused feature map $f_{\text {fused}}$ with the excitation via channel-wise multiplication:

$$\begin{aligned} f_{\text {PET}}= & Enc_{\text {PET}} \otimes Exc_{\text {PET}} \\ f_{\text {CT}}= & Enc_{\text {CT}} \otimes Exc_{\text {CT}} \end{aligned}$$

The excited features are passed through a final set of max pooling and normalization layers to yield the final output:

$$\begin{aligned} PET_{\text {excited}}= & Norm(Pool_{\text {MP}}(f_{\text {PET}}) \\ CT_{\text {excited}}= & Norm(Pool_{\text {MP}}(f_{\text {CT}}))) \end{aligned}$$

### Anatomy-guided intermediate fusion

The extracted features from PET and CT are fused in the intermediate fusion block. The features are first pooled using average pooling and max pooling, then processed through convolution layers to reduce dimensions. The pooled features ($PET^{\text {pf}}$ and $CT^{\text {pf}}$) are represented as:

$$\begin{aligned} PET^{\text {pf}}= & Conv^F_{1,1}(Pool_{\text {MP}}(Pool_{\text {AP}}(PET_{\text {excited}}))) \\ CT^{\text {pf}}= & Conv^F_{1,1}(Pool_{\text {MP}}(Pool_{\text {AP}}(CT_{\text {excited}}))) \end{aligned}$$

Fusion is performed by element-wise addition and ReLU activation, followed by a convolution layer:

$$\begin{aligned} f_{\text {fused}} = \sigma \left( PET^{\text {pf}} \oplus CT^{\text {pf}})\right) \end{aligned}$$

Now the anatomy guidance is provided by element-wise multiplying the fused features with the excited CT features from the encoder.

$$\begin{aligned} CT_{guidance} = CT_{\text {excited}} * f_{\text {fused}} \end{aligned}$$

The fused feature map is processed by sigmoid activation ($\sigma$) and a 1x1 convolution to get the intermediate fusion decoder output:

$$\begin{aligned} Dec\_out = Norm \left( \sigma \left( Conv^F_{1,1}(CT_{guidance}) \right) \right) \end{aligned}$$

## Datasets

Data selection for training PET-CT segmentation models is difficult since most available datasets are limited to a few samples, usually in tens. Training with such data leads to over-fitting, which is a serious concern since the reported results may not be reliable^7^. Furthermore, the data is usually collected from a single equipment or collection center. Since PET-CT may have huge variations caused by varying equipment, the dataset from a single source may be unreliable. Due to these reasons, we select the dataset based on strict guidelines to avoid these problems and report reliable results. We select the dataset having the following features. First, it must be standardized and huge in size. Second, it must have different collection sources. Third, it must have manual PET-CT delineations. Based on these guidelines, the two datasets, HEad and neCK TumOR segmentation challenge (HECKTOR)^43^, and The Cancer Imaging Archive (TCIA)^44–46^ are selected. HECKTOR contains 224 samples obtained from five different sources. TCIA dataset consists of 137 samples from different sources.

Since the dataset is obtained from various sources, we apply normalization to both modalities. The CT volumes are clipped at Hounsfield Units between $[ -1024,1024 ]$. The volume is then mapped between the range $[-1, 1]$. PET volume is normalized using Z-score, i.e. $z = (x ^{\breve{\,}} \mu )/\sigma$, where $\mu$ is mean, and $\sigma$ is standard deviation. These techniques are proven to be a standard for PET-CT normalization^19,20,43^. Apart from the normalization, we have applied augmentation techniques, including horizontal and vertical flipping and rotation, since it contributes towards performance improvement^47^.

## Experiment and results

### Implementation environment

The suggested architecture is implemented using Keras, a python library. Nvidia RTX 2060 12G GPU, accelerated by CUDA 9.0, is used to train, test, and evaluate the architecture. The batch size is set to one while the learning rate is set to 0.005, which is reduced if the model stops learning for ten consecutive epochs. Adam optimizer is used to minimize the loss function.

### Performance evaluation

The performance of our 3D model is evaluated using the 3D dice similarity coefficient (DSC) and 95% Hausdorff Distance ($\hbox {HD}^{95}$). 3D DSC measures the volume overlap between computer-assisted segmentation and segmentation performed by an expert.

1 $$\begin{aligned} DSC \left( x,y \right) = 2\times \frac{\left| x \right| \bigcap \left| y \right| }{\left| x \right| +\left| y \right| } \end{aligned}$$

$\hbox {HD}^{95}$ measures the $95^{th}$ percentile of the distances between boundaries drawn by experts and computers.

2 $$\begin{aligned} HD^{95}\left( x,y \right) =P^{95}\left[ \underset{\text {i}\in \text {x} \text { j}\in \text {y}}{\text {sup inf }} E_{d}(i,j), \underset{\text {j}\in \text {y} \text { i}\in \text {x}}{\text {sup inf }} E_{d}(i,j) \right] \end{aligned}$$

where *x*, and *y* are ground truth and predicted tumor volumes. $E_{d}$ is the euclidean distance between *i*, and *j*. *sup*, and *inf* are supremum, and infimum. $P^{95}$ is the 95th percentile. Apart from DSC and ($\hbox {HD}^{95}$), we have used precision and recall as supporting evaluation metrics. We employ 2D DSC, Precision, and Recall to evaluate the performance of 2D architecture. These metrics are commonly used for performance evaluation^48,49^.

### Results

We follow the same strategy as the state-of-the-art to report and compare our results for the HECKTOR dataset. We use data from one data center as the test data and train our model on the rest. Table 1 shows each data center’s results, in terms of median, average, and random split. It is to be noted that the “fold” in the table refers to the data on which the model is tested. Furthermore, the deviation reported in the table refers to the maximum deviation in the folds and is not to be confused with the standard deviation. Similarly, the median refers to the median among all the data folds. Apart from the leave-one-center strategy, the data is randomly split into the test (20%) and training (80%) datasets, and the model is evaluated. It is observed that the random split produces slightly reduced performance compared to the leave one-center-split strategy. In the case of the TCIA dataset, we split the dataset randomly and report the results since the dataset does not mention the collection centers.The performance of the proposed model is also compared to the state-of-the-art models. The proposed model is compared to the generic modules (3D UNet^17^, Res UNet^50^, VNet^51^) as well as models designed specifically for PET-CT fusion (refer to Tables 2 and 3). Our model generates improved volumetric segmentation, represented by DSC, and improved delineated boundaries, represented by $\hbox {HD}^{95}$ score.

**Table 1:** The table presents the outcomes obtained using the leave one-center-alone of the HECKTOR dataset. The average of the 5-fold cross-validation is shown in the second last row. The last row shows the results obtained random data split strategy.

Data Center	DSC	$\hbox {HD}^{95}$	Precision	Recall
Fold 1 (CHGJ)	0.8427	2.82	0.8500	0.6368
Fold 2 (CHUS)	0.8003	2.00	0.7686	0.5892
Fold 3 (CHMR)	0.7987	2.43	0.7578	0.5922
Fold 4 (CHUM)	0.8082	2.00	0.8161	0.5800
Fold 5 (CHUP)	0.8421	2.30	0.8309	0.6466
Fold average	0.8184	2.31	0.8046	0.6079
Median ± deviation	0.8207±0.0220	2.41±0.41	0.8039 ±0.0461	0.6133±0.0333
Random split	0.8102 ±0.0125	3.02 ±0.13	0.84765±0.0343	0.5695±0.0532

**Table 2:** The table compares the suggested 3D model to state-of-the-art 3D models using the HECKTOR dataset.

Methods	DSC	$\hbox {HD}^{95}$	Prec	Rec
3D UNet (CT)^17^	0.4958	19.84	0.5674	0.2322
3D UNet (PET)	0.6547	13.9	0.6932	0.3749
3D UNet (PET-CT)	0.7129	7.38	0.7354	0.4442
VNet^51^	0.6398	–	0.7120	0.3511
CCUT-Net^29^	0.7044	4.26	0.7239	0.4353
Skip-scSE^52^	0.7541	4.95	0.7991	0.4871
UNet (SE Norm)^28^	0.7688	3.79	0.8224	0.5023
SimAM UNet^30^	0.7884	3.15	**0.8602**	0.5440
Res UNet^50^	0.7680	2.98	0.7298	0.5618
3D-Inception-ResNet^31^	0.7940	2.80	0.7950	0.5618
**Proposed**	**0.8184**	**2.31**	0.8046	**0.6079**

Significant values are in bold.

**Fig. 2:**
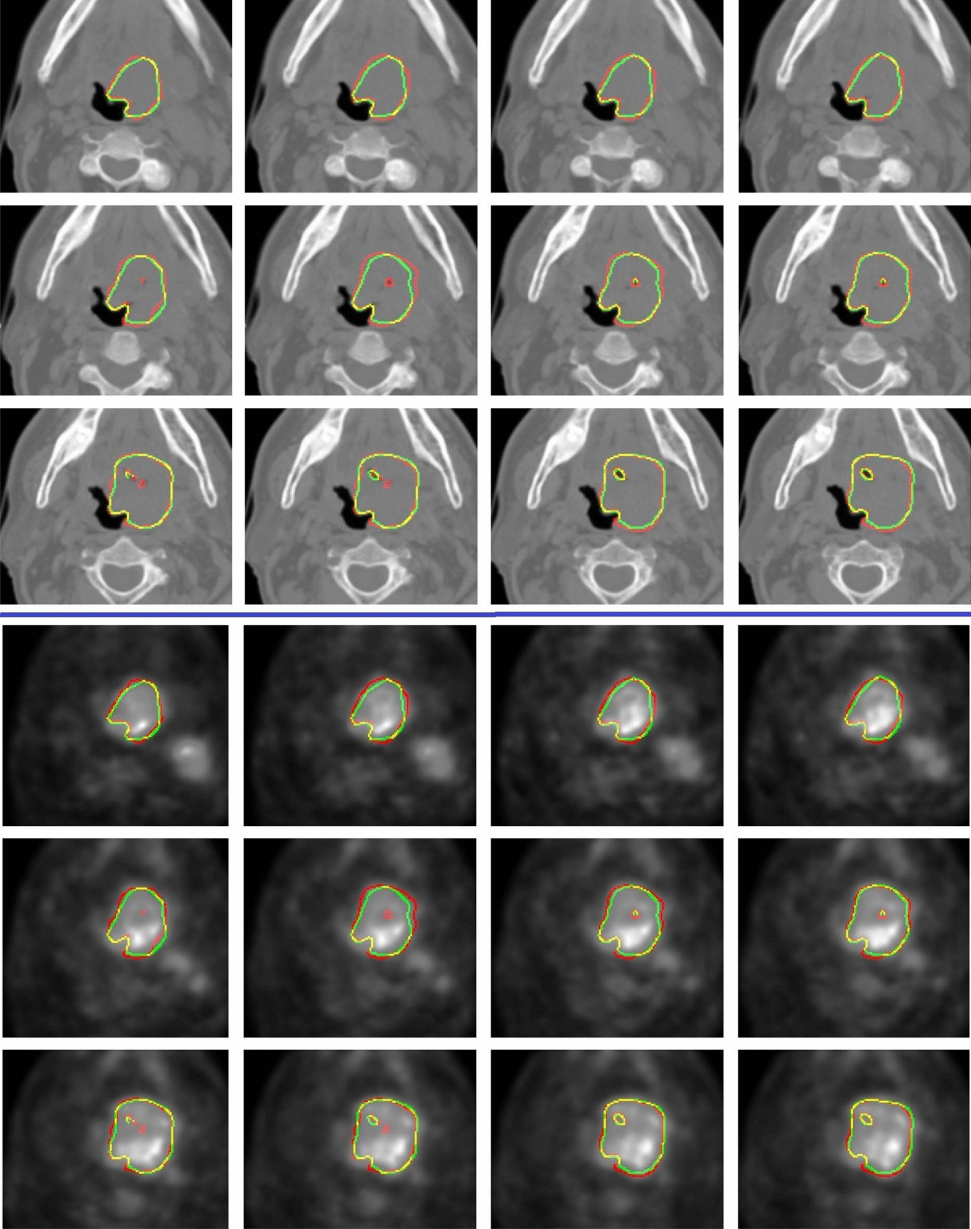
The figure depicts examples of consecutive slices from a 3D CT and PET volume, respectively, in HECKTOR dataset (CHUM center, volume 6, slice 32nd onwards.). The original tumor boundary is highlighted in red, while the predicted boundary is highlighted in green. The intersection of the original and predicted boundaries is shown in yellow.

**Fig. 3:**
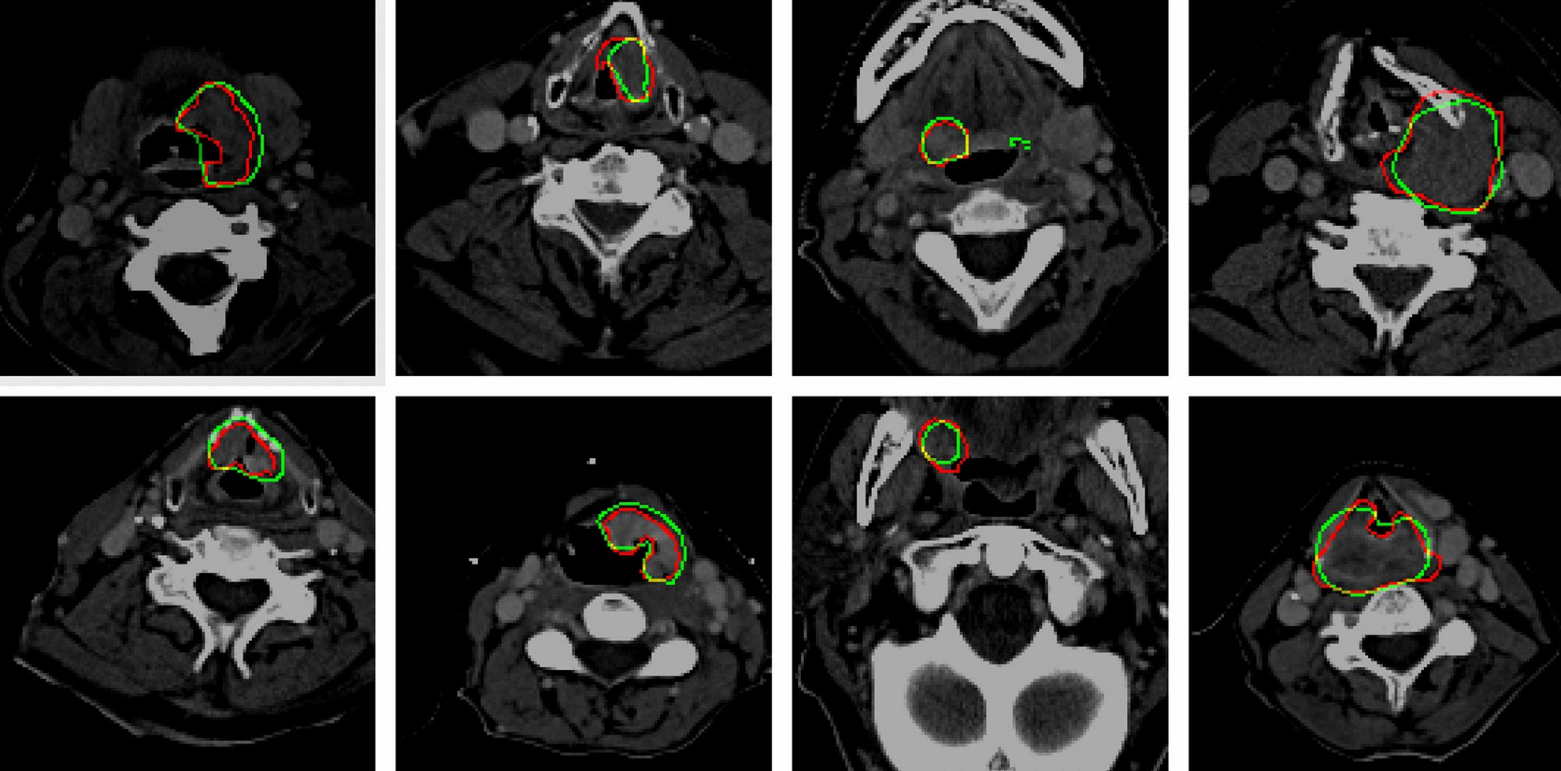
Slices from different patients in the TCIA dataset are displayed in each row of the figure. The manually drawn tumor boundary, the boundary predicted by our suggested 2D model, and the boundary overlap are represented by a different color: red, green, and yellow, respectively.

**Fig. 4:**
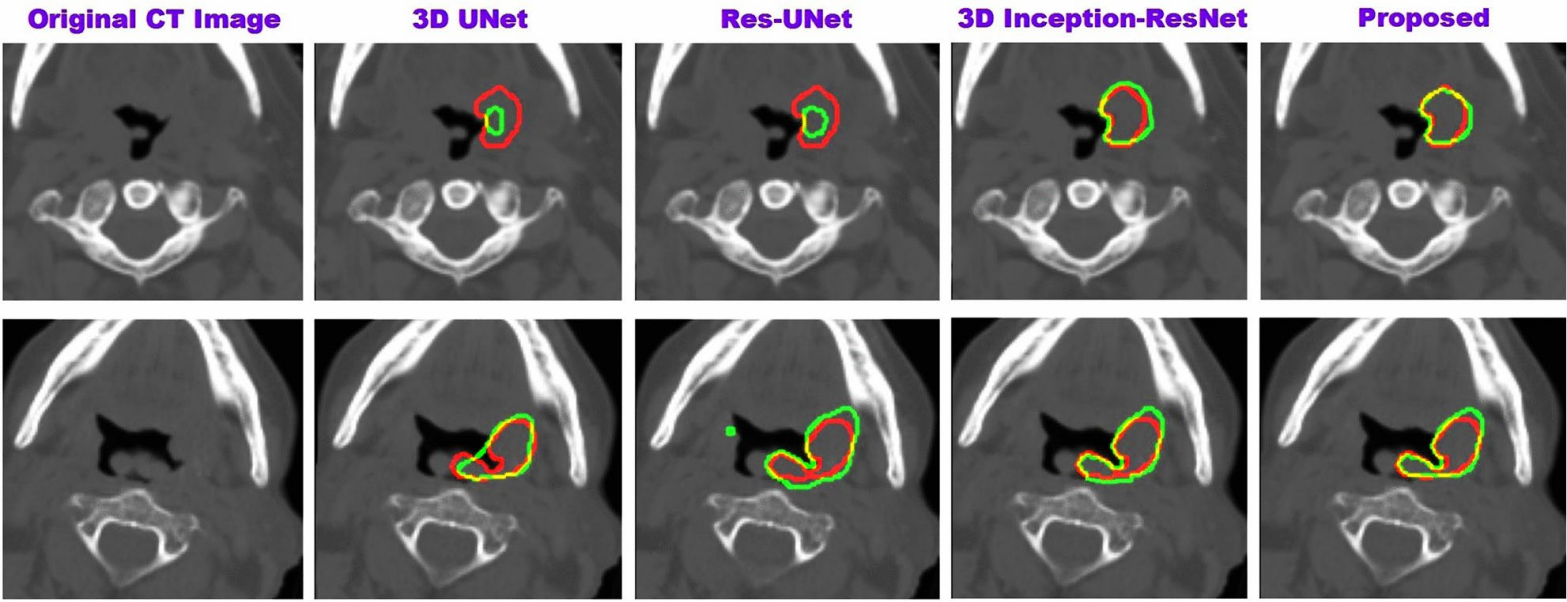
The figure shows the comparison of the proposed 3D model with the state-of-the-art methods. The manually delineated tumor boundary, the predicted boundary by our 3D model, and the boundary overlap are shown in red, green, and yellow, respectively.

**Fig. 5:**
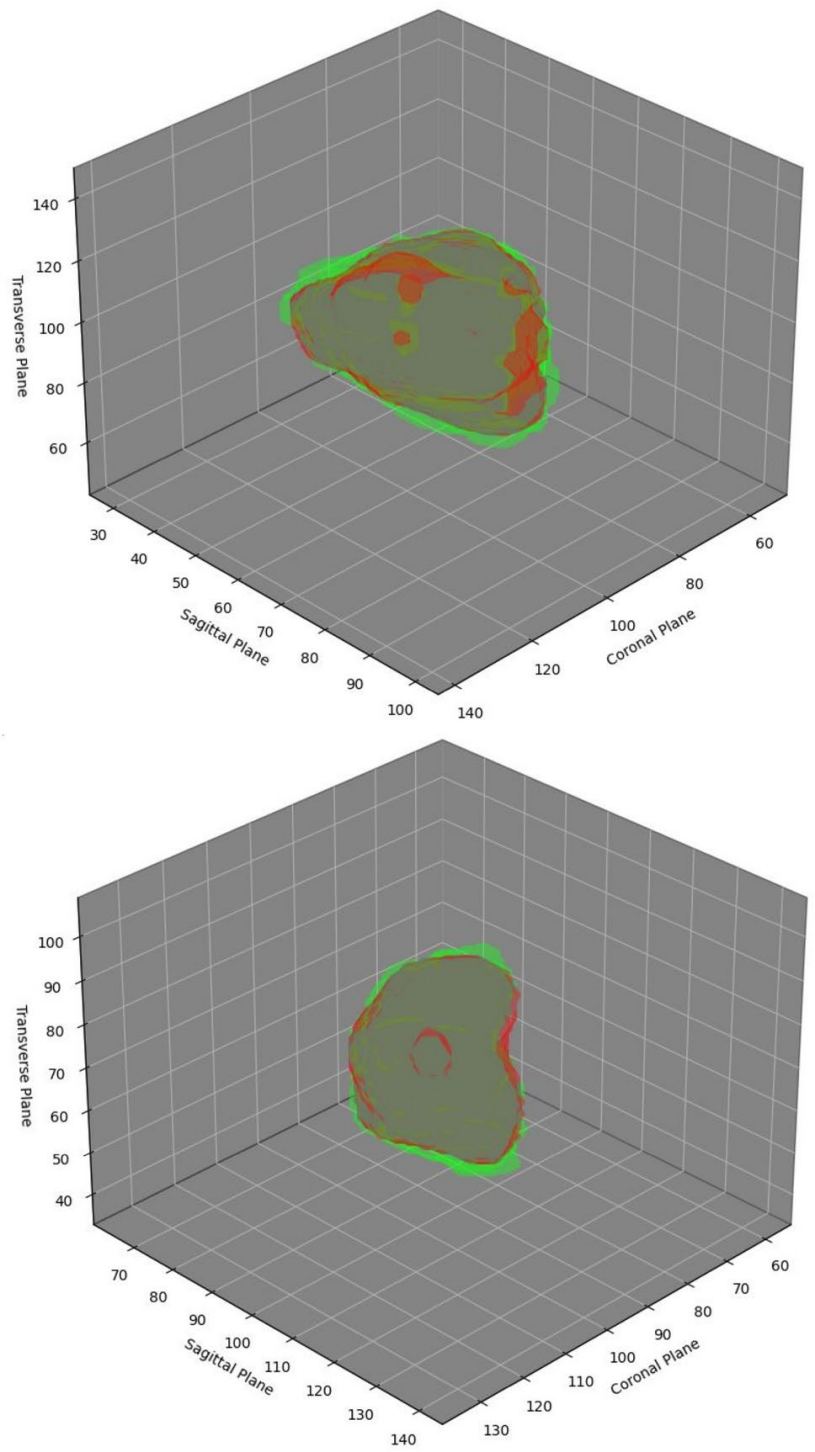
The figure shows two examples of the actual cancer (green), the segmented cancer (red), and their overlap (gray) in 3D. The axis of the figures are marked by the slice number from a 3D volume.

**Fig. 6:**
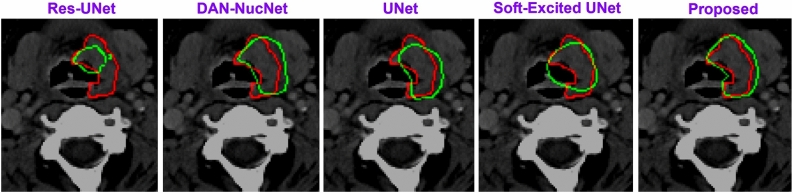
The figure shows the comparison of the proposed 2D model with some of the state-of-the-art methods. The manually delineated tumor boundary, the predicted boundary by our 2D model, and the boundary overlap are shown in red, green, and yellow, respectively.

The results obtained from the TCIA dataset are compared with the state-of-the-art in Table 3. Additionally, the 2D architecture are compared to the relevant state-of-the-art 2D models in Table 4. In Table 4, each state-of-the-art 2D model is evaluated on CT and PET individually, and then, by using early fusion. Figurative examples of segmentation generated by the 3D and 2D models are shown in Figs. 2, 3 and 5, respectively. While the 2D and 3D figurative comparison of the suggested model with the top-performing state-of-the-art models are shown in Figs. 4 and 6, respectively.

**Table 3:** The table compares the suggested 3D model to the state-of-the-art 3D models using TCIA dataset.

Methods	DSC	$\hbox {HD}^{95}$	Prec	Rec
3D UNet (CT)^17^	0.2998	11.26	0.2740	0.1299
3D UNet (PET)	0.4751	5.67	0.4154	0.2492
3D UNet (PET-CT)	0.6544	3.85	0.7750	0.3543
Skip-scSE^52^	0.7782	4.31	0.8217	0.5199
Res UNet^50^	0.7543	3.35	0.8142	0.4819
UNet (SE Norm)^28^	0.7790	**2.93**	0.8359	0.5158
**Proposed**	**0.8337**	3.16	**0.9027**	**0.5916**

Significant values are in bold.

**Table 4:** The table compares the suggested 2D model to the recent state-of-the-art 2D models using the TCIA dataset.

Methods	DSC	Prec	Rec
DIST (CT)^14^	0.4003	0.4127	0.1795
DIST (PET)	0.5045	0.4624	0.2655
DIST (PET-CT)	0.5469	0.4697	0.3139
DAN-NucNet (CT)^35^	0.4006	0.4001	0.1822
DAN-NucNet (PET)	0.4429	0.4219	0.2145
DAN-NucNet (PET-CT)	0.5363	0.4781	0.2970
ResUNet (CT)^50^	0.4045	0.3912	0.1875
ResUNet (PET)	0.4817	0.4589	0.2424
ResUNet (PET-CT)	0.5722	0.4766	0.3457
SE UNet (CT)^28^	0.3990	0.4022	0.1805
SE UNet (PET)	0.5019	0.4627	0.2625
SE UNet (PET-CT)	0.5805	0.5225	0.3359
UNet (CT)^11^	0.3910	0.3715	0.1805
UNet (PET)	0.4973	0.4328	0.2678
UNet (PET-CT)	0.6144	0.5499	0.3714
Proposed	**0.6427**	**0.6154**	**0.3847**

Significant values are in bold.

## Discussion

This section discuss various aspects of the suggested architecture in detail.

### Complexity analysis

**Fig. 7:**
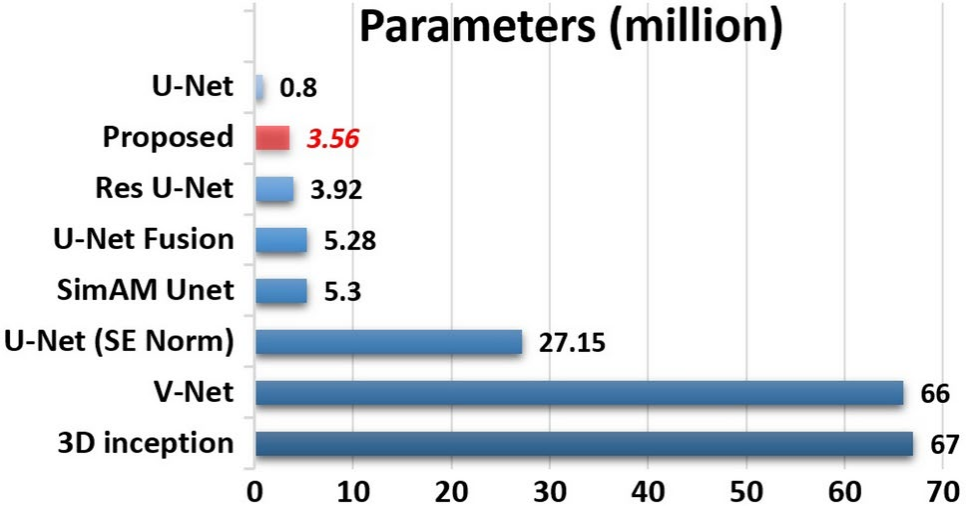
The figure compares the complexity of the suggested model to existing models in terms of trainable parameters.

Multi-modality volumetric segmentation requires enormous computational and memory resources. Therefore the complexity analysis of the 3D models is crucial. We have compared the suggested model with the state-of-the-art models (refer to Fig. 7).

The number of parameters utilized by the models are obtained using Keras’s model “summary” method. The suggested model has fewer parameters than the state-of-the-art, including VNet, 3D-Inception ResNet, and variants of UNet. The primary reason for such a low number of parameters is that the proposed model is less deep than the state-of-the-art. The state-of-the-art models are usually six to eight layers deep, which makes them extremely resource hungry. The intelligently designed decoders and encoders reduce the requirement of having more deep layers. The parameters are also reduced by the fusion of the selected features from each modality instead of fusing the whole modality.

### Ablation studies

The ablation studies in this section confirm our hypotheses about the model’s functionality. In the first study, we add individual elements and evaluate the model at each stage. We first train and test the model with only one modality, where we do not use our modality fusion mechanism. Then, we train and test the model using only a simple fusion of PET and CT. In the next stage, we fuse the PET and CT with our attentive fusion decoder. Then, we train and test the model by adding the zero layers. It is to be noted that in all the above stages, we do not use SE encoders; instead, we use encoders without SE. Finally, we train and test the model by adding the SE encoders. The results of the model at each stage is reported in Table 5. We observe that each addition to the model improves the performance significantly. The primary performance improvement is contributed by anatomy-guided intermediate fusion. The addition of zero layers and excited encoders to our model contributes further toward improved segmentation. The improvement in the DSC represents slight significant improvement in the segmentation quality. However the $HD^{95}$ shows a huge improvement representing the higher quality boundary delineation of the tumor.

**Table 5:** The table summarizes the ablation study performed using the HECKTOR dataset to determine each additional effect of the model’s sections.

Ablation	DSC	$\hbox {HD}^{95}$
CT	0.4424	14.9
PET	0.5989	8.18
PET-CT	0.7392	6.60
PET-CT attentive fusion	0.8104	3.42
PET-CT with zero layer	0.8119	3.20
PET-CT attentive fusion with SE encoder	**0.8184**	**2.31**

Significant values are in bold.

We also conducted experiments to investigate how much the data pre-processing contributes toward performance improvement. We first trained and tested our model with unprocessed data, then with normalized data (PET z-score normalization and CT clipping), and eventually with both normalization and augmentation. Table 6 summarizes the results of this ablation study. Normalization and augmentation, as expected, significantly improve segmentation.

**Table 6:** The table presents the findings of the ablation study based on data pre-processing.

Ablation	DSC	$\hbox {HD}^{95}$
Without normalization	0.6872	8.30
Normalization	0.7998	2.44
Normalization $+$ augmentation	**0.8184**	**2.31**

Significant values are in bold.

The proposed architecture guides tumor segmentation in PET volume using CT attention (anatomy awareness). In contrast to PET attention (functional awareness), this approach produces improved performance. The PET guided segmentation approach have been used in the past, however due to the lack of the spatial representations in the PET volumes/images, the segmentation quality remains low. To demonstrate this, we train and compare a model in which PET attention guides tumor segmentation to the proposed model (refer Table 7). This ablation study confirms that CT attention yields superior segmentation performance.

**Table 7:** The table depicts the ablation study, in which we individually fed both modalities to the attention block and compared their performance.

Ablation	DSC	$\hbox {HD}^{95}$
PET guided segmentation	0.7961	3.60
CT guided segmentation	**0.8184**	**2.31**

Significant values are in bold.

### Limitations

**Figure 8:**
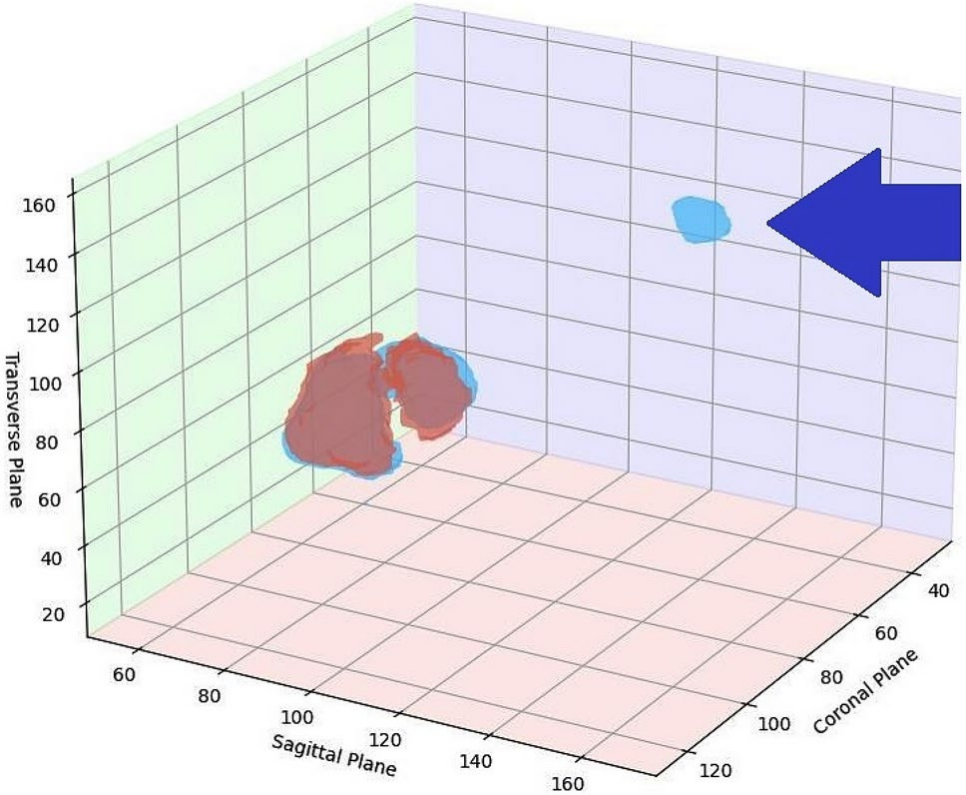
The figure shows distant metastasis in a volume that is not segmented by the proposed model..

During detailed studies, we observed that our model shows slightly improved performance for metastasis compared to the state-of-the-art. The network may still not be able to segment metastasis occasionally (Fig. 8).

The problem of miss segmentation is not related to the suggested model. It remains a major concern for the state-of-the-art models as well. While extending our framework to metastasis segmentation is an interesting direction, it falls outside the scope of this study. Future work may incorporate transfer learning, distilled learning, or other techniques to adapt the model for metastasis-related tasks. Another limitation of the model is that it may not be able to segment the early stage cancer that is near or lower to the critical mass. This can be improved by increasing the depth or resolution of the PET and CT slices. However increasing the resolution may also cause a huge increase the required computational resources.

## Conclusion

Cancer segmentation in PET-CT is critical for computer-assisted treatment, and radiotherapy planning. Existing models rely primarily on the fusion of PET-CT modalities at the decision level (late fusion) or the modality level (early fusion). Both of these techniques have disadvantages that limit segmentation performance. These methods cannot independently learn complementary features of CT and PET modalities. Furthermore, they utilize more resources and cannot use shallow inter-modality features. In this work, we employ novel zero layers, integrate excitation into our encoders that separately extract features from each modality, and fuse these modalities via anatomy-guided attentive intermediate fusion. We design the encoders by energizing them with spatial features from each modality, thus improving the spatial representations within the modalities. In addition, we introduce a zero layer that learns normalization compared to the state-of-the-art, which uses fixed normalizations. We demonstrate that our approach addresses the issues caused by early and late fusion. We show that the suggested architecture is less deep and, thus, more lightweight than existing models. We demonstrate the superiority of the suggested model through extensive results and ablation studies. Compared to the state-of-the-art, we achieved the highest DSC of 0.8184 and $\hbox {HD}^{95}$ score of 2.31. Future research could focus on improving performance for distant metastasis. Furthermore, the model could be extended to include other modalities.

## Data Availability

The two publicly available datasets, HEad and neCK TumOR segmentation challenge (HECKTOR), and The Cancer Imaging Archive (TCIA) are used in the study.

## References

[1] Kao, Y.-S. & Yang, J. Deep learning-based auto-segmentation of lung tumor PET/CT scans: A systematic review. *Clin. Transl. Imaging* **10**, 217–223. 10.1007/s40336-022-00482-z (2022).

[2] Yu, Q., Xia, Y., Xie, L., Fishman, E. K. & Yuille, A. L. Thickened 2D networks for efficient 3D medical image segmentation. 10.48550/ARXIV.1904.01150 (2019).

[3] Xia, Y. et al. Bridging the gap between 2D and 3D organ segmentation with volumetric fusion net. In Medical Image Computing and Computer Assisted Intervention–MICCAI 2018: 21st International Conference, Granada, Spain, September 16–20, 2018, Proceedings, Part IV 11, pp. 445–453. Springer International Publishing. 10.1007/978-3-030-00937-3_51 (2018).

[4] Song, Q. et al. Optimal co-segmentation of tumor in PET-CT images with context information. *IEEE Trans. Med. Imaging* **32**, 1685–1697. 10.1109/tmi.2013.2263388 (2013). 23693127 10.1109/TMI.2013.2263388 PMC3965345

[5] Bagci, U. et al. Joint segmentation of anatomical and functional images: Applications in quantification of lesions from PET, PET-CT, MRI-PET, and MRI-PET-CT images. *Med. Image Anal.* **17**, 929–945. 10.1016/j.media.2013.05.004 (2013). 23837967 10.1016/j.media.2013.05.004 PMC3795997

[6] Aygün, M., Şahin, Y. H. & Ünal, G. Multi modal convolutional neural networks for brain tumor segmentation. 10.48550/ARXIV.1809.06191 (2018).

[7] Zhou, T., Ruan, S. & Canu, S. A review: Deep learning for medical image segmentation using multi-modality fusion. *Array* **3–4**, 100004. 10.1016/j.array.2019.100004 (2019).

[8] Zhao, H., Shi, J., Qi, X., Wang, X. & Jia, J. Pyramid scene parsing network. In *Proceedings of the IEEE Conference on Computer Vision and Pattern Recognition*. 2881–2890. 10.48550/arXiv.1612.01105 (2017).

[9] Yuan, Y., Chao, M. & Lo, Y.-C. Automatic skin lesion segmentation using deep fully convolutional networks with Jaccard distance. *IEEE Trans. Med. Imaging* **36**, 1876–1886. 10.1109/tmi.2017.2695227 (2017). 28436853 10.1109/TMI.2017.2695227

[10] He, K., Zhang, X., Ren, S. & Sun, J. Deep residual learning for image recognition. In *Proceedings of the IEEE Conference on Computer Vision and Pattern Recognition*. 770–778 (2016).

[11] Ronneberger, O., Fischer, P. & Brox, T. U-Net: Convolutional networks for biomedical image segmentation. In Medical image computing and computer-assisted intervention–MICCAI 2015: 18th international conference, Munich, Germany, October 5–9, 2015, proceedings, part III 18, pp. 234–241. Springer international publishing. 10.1007/978-3-319-24574-4_28 (2015).

[12] Zhou, Z., Siddiquee, M. M. R., Tajbakhsh, N. & Liang, J. UNet++: A nested U-Net architecture for medical image segmentation. In Deep learning in medical image analysis and multimodal learning for clinical decision support: 4th international workshop, DLMIA 2018, and 8th international workshop, ML-CDS 2018, held in conjunction with MICCAI 2018, Granada, Spain, September 20, 2018, proceedings 4, pp. 3–11. Springer International Publishing. 10.1007/978-3-030-00889-5_1 (2018). 10.1007/978-3-030-00889-5_1 PMC7329239 32613207

[13] Dubost, F. *et al.* GP-Unet: Lesion detection from weak labels with a 3D regression network. In International Conference on Medical Image Computing and Computer-Assisted Intervention, pp. 214–221. Cham: Springer International Publishing. 10.1007/978-3-319-66179-7_25 (2017).

[14] Naylor, P., Lae, M., Reyal, F. & Walter, T. Segmentation of nuclei in histopathology images by deep regression of the distance map. *IEEE Trans. Med. Imaging* **38**, 448–459. 10.1109/tmi.2018.2865709 (2019). 30716022 10.1109/TMI.2018.2865709

[15] Li, X. et al. H-DenseUNet: Hybrid densely connected UNet For liver and tumor segmentation from CT volumes. *IEEE Trans. Med. Imaging* **37**, 2663–2674. 10.1109/tmi.2018.2845918 (2018). 29994201 10.1109/TMI.2018.2845918

[16] Zhang, Y., Yuan, L., Wang, Y. & Zhang, J. SAU-Net: Efficient 3D spine MRI segmentation using inter-slice attention (2020).

[17] Özgün Çiçek, Abdulkadir, A., Lienkamp, S. S., Brox, T. & Ronneberger, O. 3D U-Net: Learning dense volumetric segmentation from sparse annotation. 10.1007/978-3-319-46723-8_49 (2016).

[18] Tyagi, S., Talbar, S. N. & Mahajan, A. A novel approach of lung tumor segmentation using a 3D deep convolutional neural network. 10.4018/978-1-7998-7709-7.ch001 (2022).

[19] Bourigault, E., McGowan, D. R., Mehranian, A. & Papież, B. W. Multimodal PET/CT tumour segmentation and prediction of progression-free survival using a full-scale UNET with attention. 10.48550/ARXIV.2111.03848 (2021).

[20] Iantsen, A., Visvikis, D. & Hatt, M. Squeeze-and-excitation normalization for automated delineation of head and neck primary tumors in combined PET and CT images. 10.1007/978-3-030-67194-5_4 (2021).

[21] Yuan, Y. Automatic head and neck tumor segmentation in PET/CT with scale attention network. 10.1007/978-3-030-67194-5_5 (2021).

[22] Li, L., Zhao, X., Lu, W. & Tan, S. Deep learning for variational multimodality tumor segmentation in PET/CT. *Neurocomputing* **392**, 277–295. 10.1016/j.neucom.2018.10.099 (2020). 32773965 10.1016/j.neucom.2018.10.099 PMC7405839

[23] Jiang, J. et al. Multiple resolution residually connected feature streams for automatic lung tumor segmentation from CT images. *IEEE Trans. Med. Imaging* **38**, 134–144. 10.1109/tmi.2018.2857800 (2019). 30040632 10.1109/TMI.2018.2857800 PMC6402577

[24] Gan, W. et al. Automatic segmentation of lung tumors on CT images based on a 2D and 3D hybrid convolutional neural network. *Br. J. Radiol.* **94**, 20210038. 10.1259/bjr.20210038 (2021). 34347535 10.1259/bjr.20210038 PMC9328064

[25] Zhang, Y., Liao, Q., Ding, L. & Zhang, J. Bridging 2D and 3D segmentation networks for computation efficient volumetric medical image segmentation: An empirical study of 2.5D solutions. 10.48550/ARXIV.2010.06163 (2020). 10.1016/j.compmedimag.2022.102088 35780703

[26] Fu, X., Bi, L., Kumar, A., Fulham, M. & Kim, J. Multimodal spatial attention module for targeting multimodal PET-CT lung tumor segmentation. *IEEE J. Biomed. Health Inform.* **25**, 3507–3516. 10.1109/jbhi.2021.3059453 (2021). 33591922 10.1109/JBHI.2021.3059453

[27] Kamnitsas, K. et al. Ensembles of multiple models and architectures for robust brain tumour segmentation. 10.1007/978-3-319-75238-9_38 (2018).

[28] Xie, J. & Peng, Y. The head and neck tumor segmentation based on 3D U-Net. 10.1007/978-3-030-98253-9_8 (2022).

[29] Wang, J., Peng, Y., Guo, Y., Li, D. & Sun, J. CCUT-Net: Pixel-wise global context channel attention UT-Net for head and neck tumor segmentation. 10.1007/978-3-030-98253-9_2 (2022).

[30] Liu, T., Su, Y., Zhang, J., Wei, T. & Xiao, Z. 3D U-Net applied to simple attention module for head and neck tumor segmentation in PET and CT images. 10.1007/978-3-030-98253-9_9 (2022).

[31] Qayyum, A., Benzinou, A., Mazher, M., Abdel-Nasser, M. & Puig, D. Automatic segmentation of head and neck H and N primary tumors in PET and CT images using 3D-inception-ResNet model. 10.1007/978-3-030-98253-9_4 (2022).

[32] Sreevidya, P., Veni, S. & Murthy, O. V. R. Elder emotion classification through multimodal fusion of intermediate layers and cross-modal transfer learning. *Signal Image Video Process.* **16**, 1281–1288. 10.1007/s11760-021-02079-x (2022). 35069919 10.1007/s11760-021-02079-x PMC8763433

[33] Li, Y. et al. Multimodal information fusion for glaucoma and DR classification. 10.48550/ARXIV.2209.00979 (2022).

[34] Bhat, S. & Koundal, D. Multi-focus image fusion techniques: A survey. *Artif. Intell. Rev.* **54**, 5735–5787. 10.1007/s10462-021-09961-7 (2021).

[35] Ahmad, I., Xia, Y., Cui, H. & Islam, Z. U. DAN-NucNet: A dual attention based framework for nuclei segmentation in cancer histology images under wild clinical conditions. *Expert Syst. Appl.* **213(Part A)**, 118945. 10.1016/j.eswa.2022.118945 (2023).

[36] Li, H., Wu, X.-J. & Durrani, T. NestFuse: An infrared and visible image fusion architecture based on nest connection and spatial/channel attention models. *IEEE Trans. Instrum. Meas.* **69**, 9645–9656. 10.1109/tim.2020.3005230 (2020).

[37] Yu, N., Li, J. & Hua, Z. Attention based dual path fusion networks for multi-focus image. *Multimed. Tools Appl.* **81**, 10883–10906. 10.1007/s11042-022-12046-4 (2022).

[38] Bi, L. et al. Recurrent feature fusion learning for multi-modality PET-CT tumor segmentation. *Comput. Methods Prog. Biomed.* **203**, 106043. 10.1016/j.cmpb.2021.106043 (2021). 10.1016/j.cmpb.2021.106043 33744750

[39] Xiao, N. et al. PET and CT image fusion of lung cancer with siamese pyramid fusion network. *Front. Med.* **9**. 10.3389/fmed.2022.792390 (2022). 10.3389/fmed.2022.792390 PMC9010034 35433720

[40] Chin, M. et al. Self-normalization for a 1-mm3 resolution clinical pet system using deep learning. *Phys. Med. Biol.*10.1088/1361-6560/ad69fb (2024). 39084640 10.1088/1361-6560/ad69fb

[41] Huang, L., Xia, W., Zhang, B., Qiu, B. & Gao, X. MSFCN-multiple supervised fully convolutional networks for the osteosarcoma segmentation of CT images. *Comput. Methods Prog. Biomed.* **143**, 67–74. 10.1016/j.cmpb.2017.02.013 (2017). 10.1016/j.cmpb.2017.02.013 28391820

[42] Ren, J., Li, M. & Korreman, S. Sine wave normalization for deep learning-based tumor segmentation in CT/PET imaging. arXiv: 2409.13410. 10.48550/arXiv.2409.13410 (2024).

[43] Oreiller, V. et al. Head and neck tumor segmentation in PET/CT: The HECKTOR Challenge. *Med. Image Anal.* **77**, 102336. 10.1016/j.media.2021.102336 (2022). 35016077 10.1016/j.media.2021.102336

[44] Wee, L. & Dekker, A. Data from head-neck-radiomics-HN1. 10.7937/TCIA.2019.8KAP372N (2019).

[45] Aerts, H. J. W. L. et al. Decoding tumour phenotype by noninvasive imaging using a quantitative radiomics approach. *Nat. Commun.* **5**. 10.1038/ncomms5006 (2014). 10.1038/ncomms5006 PMC4059926 24892406

[46] Clark, K. et al. The cancer imaging archive (TCIA): Maintaining and operating a public information repository. *J. Digit. Imaging* **26**, 1045–1057. 10.1007/s10278-013-9622-7 (2013). 23884657 10.1007/s10278-013-9622-7 PMC3824915

[47] Sobirov, I., Nazarov, O., Alasmawi, H. & Yaqub, M. Automatic segmentation of head and neck tumor: How powerful transformers are? 10.48550/ARXIV.2201.06251 (2022).

[48] Gudi, S. et al. Interobserver variability in the delineation of gross tumour volume and specified organs-at-risk during IMRT for head and neck cancers and the impact of FDG-PET/CT on such variability at the primary site. *J. Med. Imaging Radiat. Sci.* **48**, 184–192. 10.1016/j.jmir.2016.11.003 (2017). 31047367 10.1016/j.jmir.2016.11.003

[49] Moe, Y. M. et al. Deep learning for automatic tumour segmentation in PET/CT images of patients with head and neck cancers. 10.48550/ARXIV.1908.00841 (2019).

[50] Naser, M. A. et al. Head and neck cancer primary tumor auto segmentation using model ensembling of deep learning in PET/CT images. 10.1007/978-3-030-98253-9_11 (2022). 10.1007/978-3-030-98253-9_11 PMC8991449 35399869

[51] Milletari, F., Navab, N. & Ahmadi, S.-A. V-net: Fully convolutional neural networks for volumetric medical image segmentation. 10.48550/ARXIV.1606.04797 (2016).

[52] Biase, A. D. et al. Skip-SCSE multi-scale attention and co-learning method for oropharyngeal tumor segmentation on multi-modal PET-CT images. 10.1007/978-3-030-98253-9_10 (2022).

